# Molecular basis for the wide range of affinity found in Csr/Rsm protein–RNA recognition

**DOI:** 10.1093/nar/gku141

**Published:** 2014-02-21

**Authors:** Olivier Duss, Erich Michel, Nana Diarra dit Konté, Mario Schubert, Frédéric H.-T. Allain

**Affiliations:** Institute of Molecular Biology and Biophysics, ETH Zürich, 8093 Zürich, Switzerland

## Abstract

The carbon storage regulator/regulator of secondary metabolism (Csr/Rsm) type of small non-coding RNAs (sRNAs) is widespread throughout bacteria and acts by sequestering the global translation repressor protein CsrA/RsmE from the ribosome binding site of a subset of mRNAs. Although we have previously described the molecular basis of a high affinity RNA target bound to RsmE, it remains unknown how other lower affinity targets are recognized by the same protein. Here, we have determined the nuclear magnetic resonance solution structures of five separate GGA binding motifs of the sRNA RsmZ of *Pseudomonas fluorescens* in complex with RsmE. The structures explain how the variation of sequence and structural context of the GGA binding motifs modulate the binding affinity for RsmE by five orders of magnitude (∼10 nM to ∼3 mM, *K*_d_). Furthermore, we see that conformational adaptation of protein side-chains and RNA enable recognition of different RNA sequences by the same protein contributing to binding affinity without conferring specificity. Overall, our findings illustrate how the variability in the Csr/Rsm protein–RNA recognition allows a fine-tuning of the competition between mRNAs and sRNAs for the CsrA/RsmE protein.

## INTRODUCTION

Regulation by small non-coding RNAs (sRNAs) is crucial for orchestrating global changes in bacterial gene expression (1–3). The best studied small regulatory RNAs in bacteria function by Hfq chaperone-assisted base pairing with target messenger RNAs (mRNAs) (4,5). They often work by binding to the ribosome binding site (RBS), thereby repressing mRNA translation and through recruiting RNase E via Hfq, they can target the mRNA for degradation (6). Another important group of sRNAs do not act on mRNAs directly but function by sequestering the CsrA-type protein from the RBS of a subset of mRNAs and thus, activate translation initiation (1,7,8).

The Csr system has been characterized as regulating global pathways involved in central carbon metabolism, cell motility, biofilm formation, quorum sensing, the production of extracellular products and is viewed as the most important post-transcriptional regulator of bacterial pathogenesis (7–10). Homologues of the CsrA protein have been found to be widely distributed among bacteria (encoded by ∼75% of all species) (9). The orthologues of CsrA (carbon storage regulator), such as RsmA or RsmE (regulator of secondary metabolism), have high sequence identity and similarity for all protein amino acid side-chains contributing to RNA recognition (11). Remarkably, even the very recently identified orthologues RsmN/RsmF protein from *Pseudomonas aeruginosa* has a conserved RNA recognition surface, despite a distinctly different polypeptide fold when compared with the domain-swapped dimeric structure of the CsrA/RsmE homologues (12,13). The 15 kDa CsrA/RsmE homo-dimer is able to bind, using two identical binding sites, to RNA sequences containing a central GGA motif flanked by additional nucleotides which are also bound (11,14). In general, the 5′-UTR of the CsrA/RsmE-regulated mRNAs contain between one and several GGA motifs that bind CsrA/RsmE with different affinities (10,15–17). Often, a high affinity GGA motif overlaps directly with the RBS and can bind CsrA/RsmE efficiently, preventing the docking of the RBS to the 30S ribosomal subunit and thus inhibiting translation initiation. It is also possible that the GGA motif overlapping the RBS has only low affinity for CsrA/RsmE and is therefore not able to efficiently repress ribosome loading (16). However, other GGA motifs located upstream of the RBS with high affinity for CsrA can recruit the homo-dimeric protein, thereby increasing its local concentration to enable cooperative binding to the RBS and efficient translation repression (16). The de-repressor sRNAs also contain several GGA binding motifs, typically located in hairpin loops (14,18–21), also in single-stranded regions or even buried within secondary structures.

We have previously solved the solution nuclear magnetic resonance (NMR) structure of a stem–loop encompassing the RBS of the *Pseudomonas fluorescens* CHA0 *hcnA* mRNA in complex with RsmE (11). The structure shows how the guanine and adenine bases of the **A**C**GGA**U hexanucleotide loop of the 20 nt stem–loop RNA (20 nt-RBS RNA) are specifically recognized by the protein backbone and thus, the shape of the protein, while the RNA stem is semi-specifically recognized on its major groove side by protein side-chains. Although this structure demonstrates the molecular basis for the CsrA/RsmE protein recognition by a specific GGA motif, which is close to the high affinity SELEX-derived consensus sequence (14), it is unclear how other GGA motifs in different sequence and structural contexts would be recognized by the CsrA/RsmE protein. To deepen the understanding of CsrA/RsmE recognition by the diverse class of GGA binding motifs, we solved the solution structures of five different GGA motifs, all located within the sRNA RsmZ of *P. fluorescens*, in complex with the RsmE protein. We show how the same protein can recognize different RNA targets by plasticity of both protein and RNA structure. The sequence and structural context of a GGA motif allows modulation in the affinity by more than five orders of magnitude. Overall, we provide the framework for predicting the binding affinity of a certain GGA motif for the CsrA/RsmE protein according to its sequence and structural context. Using this framework, we can successfully predict the effect of sRNA mutations on translation activation by the *P. fluorescens* sRNA RsmX *in vitro* and rationalize the sequential binding of RsmE to the 5′-UTR of the *P. fluorescens hcnA* mRNA.

## MATERIALS AND METHODS

### Protein and RNA sample preparation

The RsmE protein homo-dimer was expressed recombinantly and purified with a C-terminal histidine tag as reported previously (11). The RNA was obtained by *in vitro* transcription from double-stranded DNA templates using T7 polymerase and was subsequently purified by denaturing high-performance liquid chromatography (HPLC) followed by butanol extraction as reported earlier (22,23). Due to its small size, the 9 nt-GGA_39__–__41_ RNA was first transcribed as a longer precursor RNA, which was subsequently cut into smaller fragments by sequence-specific RNase H cleavage. Protein–RNA complexes used for NMR experiments were prepared by mixing the protein and RNA at a 1:1 ratio with a typical concentration of 1 mM in a buffer containing 30 mM NaCl and 50 mM potassium phosphate at pH 7.2.

### NMR spectroscopy

NMR spectra were acquired at 313 K except for 2D ^1^H-^1^H NOESY experiments in H_2_O, which were performed at 283 K to observe RNA imino protons in stem regions of RNA hairpins. The spectra were recorded on Bruker Avance III 500, 600, 700 or 900 MHz spectrometers equipped with a cryo probe. All spectra were processed with Topspin 2.1 or 3.0 and analysed in Sparky 3.0. The ^1^H, ^13^C and ^15^N chemical shifts of the RsmE protein in complex with the 5 RNA targets were assigned by standard methods (24,25). The RNA imino protons were assigned with 2D ^1^H-^1^H NOESY experiments (τ_m_ = 250 ms) in 95% H_2_O/5% D_2_O (v/v) at 283 K. Non-exchangeable RNA proton resonances were assigned using 2D ^1^H-^1^H NOESY (τ_m_ = 150 ms), ^1^H-^1^H TOCSY, ^1^H-^13^C HSQC, 3D ^13^C-edited NOESY (τ_m_ = 150 ms) and 3D HC(C)H TOCSY spectra in D_2_O with samples, in which the RsmE protein was only ^15^N-labelled and the RNA nucleotide-specifically ^13^C,^15^N-labelled (with either A, U or G, C labelled) (25). Due to severe spectral overlap of the RNA resonances for SL3 in complex with RsmE, a third cytosine-only ^13^C,^15^N-labelled RNA in complex with only ^15^N-labelled RsmE protein was prepared. For the 9 nt-GGA_39-41_ RNA in complex with RsmE, it was sufficient to isotopically label the RNA uniformly for full assignment. The intra- and intermolecular nuclear Overhauser effect (NOE) signals were assigned based on 2D ^1^H-^1^H nuclear Overhauser enhancement spectroscopy (NOESY) (τ_m_ = 150 ms), 3D ^13^C-edited NOESY (τ_m_ = 150 ms) and 3D (F1-edited, F3-filtered) NOESY spectra (τ_m_ = 150 ms) (25) of samples in which either the protein was ^13^C,^15^N-labelled and the RNA unlabelled, or the protein only ^15^N-labelled and the RNA nucleotide-specific ^13^C,^15^N-labelled as described above. The NOEs were semi-quantitatively classified according to their intensities in the 2D and 3D NOESY spectra. Hydrogen bonding distance restraints were based on the observation of an imino resonance of the corresponding base pair. Angle restraints of the sugar pucker conformations were extracted from ^1^H-^1^H TOCSY spectra. Protein torsion angle restraints were obtained from TALOS + (26).

The heteronuclear ^1^H-^15^N NOE experiments were recorded in an interleaved fashion, recording alternatively one increment for the NOE or the reference spectrum (27). The relaxation delay was 2 s and the ^1^H pre-saturation delay 3 s for the NOE experiment, while a 5 s relaxation delay was used in the reference experiment.

### Structure calculation and refinement

Preliminary structures were generated by a simulated annealing protocol using the CYANA package (28) including manually assigned NOE distance, torsion angle and hydrogen bond constraints as summarized in Supplementary Table S1. A total of 999 structures were generated starting from random coil RNA and protein chains using 20 000 simulated annealing steps. An ensemble of the 50 lowest target energy structures was selected and further refined in AMBER 9.0 (29,30). The complex was refined in implicit solvent using the same distance, torsion angle and hydrogen bond constraints as used in the CYANA simulated annealing protocol. The force field ff99 (31) was used along with the generalized Born model (32) to mimic the solvent. The 20 lowest energy structures were selected. The structural statistics are presented in Supplementary Table S1.

### Isothermal titration calorimetry

The isothermal titration calorimetry (ITC) binding experiments were conducted on a VP-ITC instrument from MicroCal. The calorimeter was calibrated according to the manufacturer’s instructions. The RNA and the protein samples were dialysed against the same buffer batch (300 mM NaCl, 50 mM potassium phosphate at pH 8.0). Concentrations were determined after dialysis using optical density absorbance at 260 and 280 nM for RNA or protein, respectively. The RNA (syringe, 100–600 µM concentration) was titrated into the RsmE protein (cell, 5–30 µM dimer concentration). ITC binding experiments were performed at 298 K and typically consisted of 30–40 injections of 4–10 µl with an injection speed of 2 s/µl and a 5 min interval between additions. The stirring rate was 307 rpm. All measurements were repeated at least twice. Using Origin 7.0, the raw data was integrated, corrected for non-specific heats and analysed according to a one-site or two-site model. The RNA sequences of all the constructs are summarized in Supplementary Table S2.

### Binding affinity determination by NMR spectroscopy

In the slow exchange regime on the NMR timescale, the resonances corresponding to the free and bound macromolecules are simultaneously present as two separated peaks if their chemical environments change upon complex formation. Their relative integrals directly relate to the fraction of free and bound macromolecule (protein or RNA) present in the sample. Knowing the total concentration of macromolecules (protein and RNA), the dissociation constant can directly be determined using the equation:
(1)
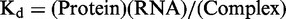



As only a single well-separated resonance (imino of the second G in the G**G**A motif forming an intermolecular hydrogen bond) is observable for the unlabelled RNA in complex, the dissociation constants with their associated errors for the hcnA-GGA#1 and hcnA-GGA#2 RNAs binding to RsmE (Figure 6) were determined using several amide resonance pairs of free and bound protein at a total concentration of protein and RNA of 0.54 mM.

### Cell-free translation assay

The 5′-UTR of the *hcnA* gene was subcloned into the vector pIVEX1.3-CAT (Roche) directly upstream of the chloramphenicol acetyl-transferase (CAT) open reading frame, yielding the vector pCFX100, which was amplified using a plasmid maxi prep kit (Macherey-Nagel). S30 cell extract was obtained from *Escherichia coli* BL21 (DE3) Star cells, which were genetically modified by introducing a C-terminal (His)_6_-tag in the *csrA* gene following the procedure by Datsenko and Wanner (33), resulting in the strain *E. coli* BL21 (DE3) Star *csrA::(His)_6_*. In addition to the previously described extract preparation protocol (34), the endogenous *csrA* protein was removed by passing the cell lysate directly over Ni-NTA beads. The obtained S30 extract devoid of *csrA* was then used for *in-vitro* translation of the CAT reporter gene from pCFX100 according to the previously described protocol (34). The RsmX sRNA was prepared by *in vitro* transcription from a linearized plasmid and purified by denaturing HPLC as described previously (22,23).

Analytical scale cell-free expressions (50 µl) of the pCFX100 plasmid were set up in presence of various amounts of wild-type or mutant RsmX sRNA and 100 nM dimeric RsmE protein from *P. fluorescens*. After 3 h of cell-free expression, the reaction mixture was centrifuged and placed on ice. A quantity of 5 µl of the reaction supernatant was thoroughly mixed with 495 µl dilution buffer (100 mM Tris–HCl at pH 7.8, 1 mg/ml BSA). A total of 10 µl of the diluted solution was then mixed with 990 µl CAT reaction solution (100 mM Tris–HCl at pH 7.8, 0.5 mM DTNB, 50 µM acetyl-CoA, 50 µM chloramphenicol, 1 mg/ml BSA) and the increase in absorbance at 412 nm was followed for 20 min on a Cary 300 Bio spectrophotometer. The expression levels of the reporter enzyme were derived from the slope of absorbance at 412 nm against time and were then normalized to the largest value that was obtained after complete saturation of RsmE in the reaction mixture with RsmX RNA.

## RESULTS

### Solution structures of five different GGA motifs of RsmZ bound to RsmE

**Figure 1.**
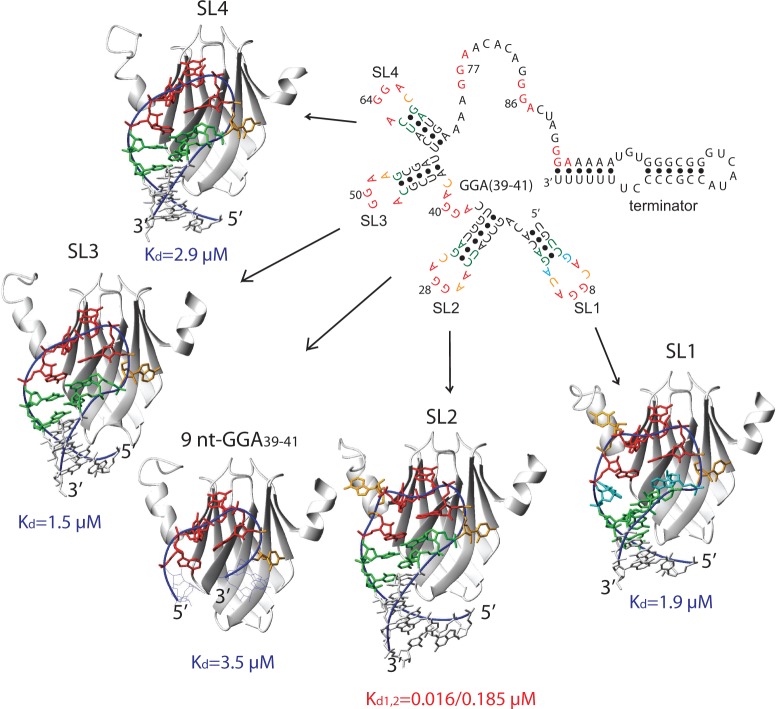
NMR solution structures of five different GGA motifs of RsmZ in complex with RsmE. The predicted secondary structure of the RsmZ sRNA and the lowest energy structure of each protein–RNA complex with its corresponding *K*_d_ (determined by ITC) are presented. The second RNA molecule binding to the homo-dimeric RsmE protein is not shown for simplicity. The conserved **A(**N)**GGA**X motif is shown in red, the CG/UA stem closing base pair in green, the looped-out nucleotides N and X in orange and the inserted nucleotides between the A(N)GGAX loop and the CG-UA stem closing base pair in SL1 are shown in cyan. The RNA nucleotides in the complex structures are for SL1: 1–16, SL2: 19–36, SL3: 43–57, SL4: 58–72 and 9 nt-GGA_39-41_: 36–44 (U36, C37, A43 and U44 are unstructured and shown in grey line representation). For SL2 binding to the homo-dimeric RsmE protein, the ITC binding data could only be fitted using a two-site binding model (both *K*_d_ values shown), whereas the binding for the other complexes could be fitted using a one-site binding model only. An overview of all ITC titration curves is presented in Supplementary Figure S2****.

The sRNA RsmZ from *P. fluorescens* CHA0 is a sRNA of 127 nt composed of four stem–loops (SLs) and a ρ-independent terminator (19). It contains eight GGA motifs that are predicted to bind RsmE (Figure 1). Here, we have solved the NMR solution structures of the four isolated stem–loops and of the single-stranded region between SL2 and SL3 of the RsmZ sRNA in complex with the RsmE protein from *P. fluorescens CHA0*. All four stem–loops of RsmZ contain a conserved A(N)GGAX motif in the loop on top of a stem closed by two base pairs, a C-G followed by a U-A, except SL3 that has a G-C instead of a U-A as penultimate stem-closing base pair (Figure 1). However, the loop length varies between 5 and 8 nt. SL3 and SL4 have the shortest loop sequence of 5 nt, in which the nucleotide N in A(N)GGAX is missing. SL2 contains 6 nt in the loop like the 20 nt-RBS RNA. The difference between the two stem–loops occurs at the nucleotides N and X and the lower part of the stem. SL1 has the longest loop (8 nt), in which two additional nucleotides are inserted between the ANGGAX sequence and the stem. The RNA sequence between SL2 and SL3 (9 nt-GGA_39-41_) is single-stranded and is also missing the nucleotide N of the consensus motif.

First, we characterized the binding of these RNAs to RsmE with NMR chemical shift titrations and ITC-binding experiments. All the protein and RNA resonances in the 4 SL complexes are in slow exchange relative to the NMR chemical shift timescale (Supplementary Figure S1). Unexpectedly, the complex with the single-stranded 9 nt-GGA_39-41_ RNA is also in slow exchange indicating a slow rate of complex dissociation (<10/s). The binding affinities differ by one to two orders of magnitude. Whereas SL2 has an affinity of 16 ± 3 and 185 ± 3 nM for the binding of the first and second RNA molecule to the dimeric RsmE protein, respectively, the other RNA targets have affinities ranging from 1.5 to 3.5 µM (Figure 1 and Supplementary Figure S2).

To understand the molecular basis for these differences in affinity, we determined the structures of the corresponding protein–RNA complexes. The high quality of the spectra allowed us to collect enough intra- and intermolecular NOEs to solve the structures of all five complexes with an RMSD of 0.75–1.2 Å. The structural statistics are shown in Supplementary Table S1. All the RNA nucleotides are well defined in the stem–loop RNA complexes (Supplementary Figure S3). In the single-stranded 9 nt-GGA_39-41_ complex structure, the nucleotides flanking the common A(N)GGAX binding motif, are not well defined and are not recognized by the RsmE protein (Supplementary Figure S3). They are flexible as can be judged from the narrower line width of their aromatic and sugar resonances. An overview of all the structures is shown in Figure 1.

### Common binding mode

The conserved **A**(N)**GGA**X motif is recognized in an identical manner in all five structures, also found in the structure of the 20 nt-RBS RNA bound to RsmE (11) (Supplementary Figure S4). As the recognition of the **A**(N)**GGA**X motif is largely achieved by many hydrogen bonds between the RNA bases and the protein backbone, the recognition is sequence specific and these bases cannot be substituted by others and still be accommodated by the given protein fold. In contrast to the core **A**(N)**GGA**X motif, the nucleotides N and X within and the nucleotides adjacent to it are variable and allow for a modulation of the binding affinity.

### Nucleotide N in A(N)GGAX contributes to binding affinity

**Figure 2.**
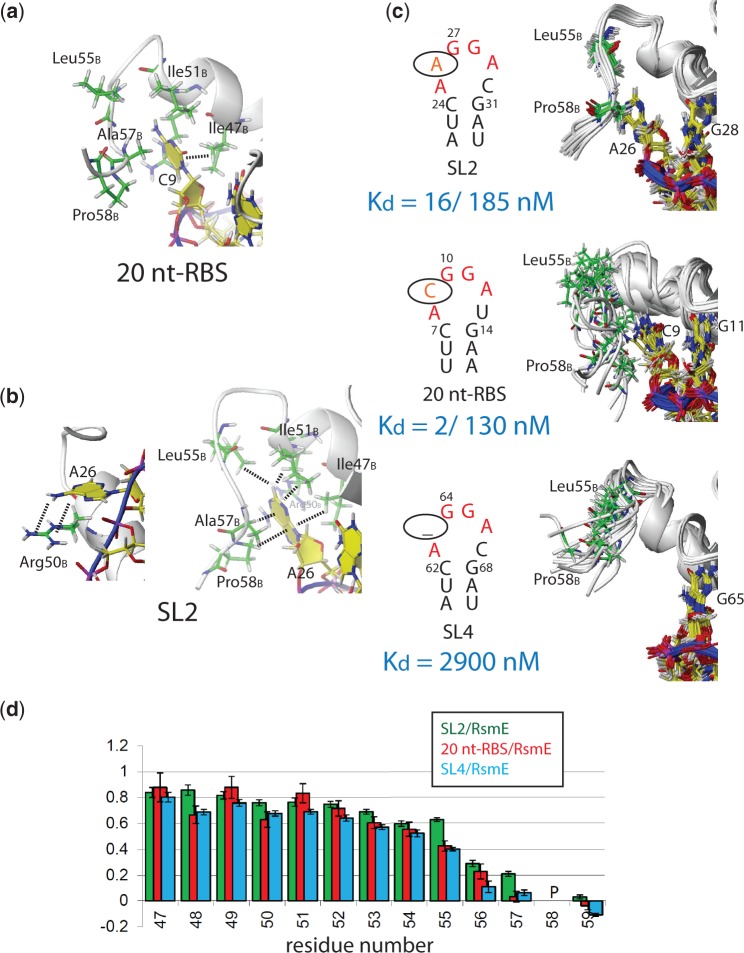
Recognition of the looped-out nucleotide N in A(**N**)GGAX. (**a** and **b**) Structural details of interactions important for recognition of the nucleotide N in 20 nt-RBS (a) and SL2 (two views) (b). (**c**) NMR structural ensemble of C-terminus of RsmE contacting A26 in SL2 (top), C9 in 20 nt-RBS (center) and no base in SL4 (bottom). The *K*_d_ values are indicated for the different RNAs. For the 20 nt-RBS and SL2, the two *K*_d_ values correspond to the first and second RNA molecule binding to the homo-dimeric RsmE protein, respectively. (**d**) ^15^N-heteronuclear NOE values of the C-terminus of RsmE in complex with SL2 (green), 20 nt-RBS (red) and SL4 (cyan). The error bars were determined according to Farrow *et al.* (35).

The presence of the looped out nucleotide N contributes to a one to three orders of magnitude increase in binding affinity (Figure 2). If the looped out residue is a cytosine as in 20 nt-RBS or SL1, hydrophobic interactions of the base with the side-chain of Ile47_B_ are present (Figure 2a). In contrast, when a larger adenine is looped-out as in SL2, more hydrophobic contacts are observed between the H2 proton and the larger surface of the purine base of adenosine A26 with the aliphatic side-chains of Ile47_B_, Arg50_B_, Ile51_B_, Leu55_B_, Ala57_B_ and Pro58_B_ (Figure 2b). Interestingly, this adenine also stacks on Arg50_B_ (Figure 2b, left); this interaction is not possible for the cytosine which is too small to stack with Arg50_B_. Obviously, all these interactions are lost if there is no base looped out as in SL3, SL4 or the ssRNA 9 nt-GGA_39-41_ (Figure 2c, bottom image), rationalizing the significant loss in affinity when the nucleotide N in A(N)GGAX is not present.

Despite the larger number of hydrophobic contacts and the stacking of adenine A26 on Arg50_B_, the affinity of SL2 is very similar to that of 20 nt-RBS for RsmE (both RNA sequences in the ANGGAX loop are identical in the region that contacts the protein except for the two looped out nucleotides N and X). A static structure allows a rationalization of the enthalpic contribution to the free energy of binding but does not provide any insight into the entropic contribution to binding, which might explain why the affinity of SL2 is not significantly higher than that of the 20 nt-RBS for RsmE. Remarkably, ITC-binding experiments show a significantly higher binding enthalpy for SL2 compared with the 20 nt-RBS RNA and SL4 (Supplementary Figure S5). However, the favourable binding enthalpy is compensated by more unfavourable binding entropy for SL2 compared with the other two RNAs.

We then addressed the question of why SL2 binding is entropically less favoured when compared to binding of SL4 and the 20 nt-RBS RNA to RsmE. For this, we recorded ^15^N-heteronuclear NOE experiments for all three complexes, which report on the protein backbone dynamics in the ps–ns timescale (36). The ^15^N-heteronuclear values show that if the looped out nucleotide base N is an adenine (SL2), the C-terminal nucleotides Leu55 to Ala57 become more rigid than when N is a cytosine (20 nt-RBS) or N is absent (SL4) (Figure 2). These results suggest that the additional hydrophobic contacts made by a looped out adenine (a gain in enthalpy) are counteracted by unfavourable entropic contributions to the free energy of binding, at least partially due to the residues in the C-terminal α-helix becoming more ordered. This supports earlier findings that conformational entropy can significantly influence the free energy of binding (37). In summary, the presence but not the identity of the looped out nucleotide N of the A**(N)**GGAX motif results in a significant gain in binding affinity for *P. fluorescens* CHA0 RsmE. However, the energetic origin of this gain depends on the identity of the base of the looped out nucleotide.

### Nucleotides located between the ANGGAX hexa-loop and the stem decrease binding affinity

**Figure 3.**
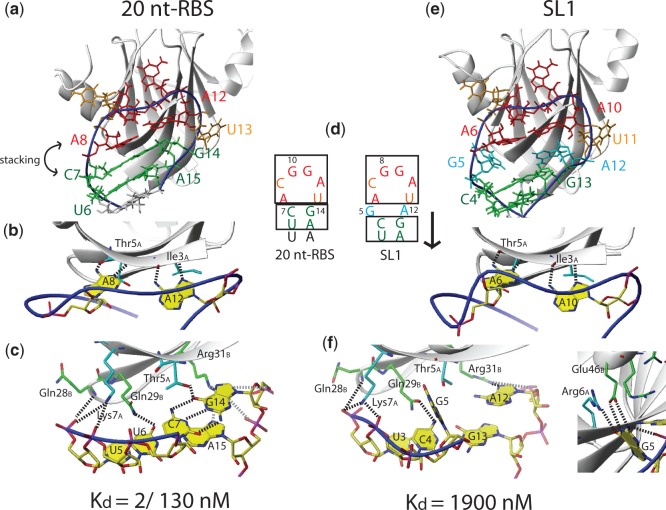
Effect of the two additional G5 and A12 nucleotides inserted between the stem and the loop in SL1. G5 and A12 in SL1 shift down the CG-UA stem closing base pairs by one register. (**d**) This disrupts hydrogen bonds that would be present between the protein and the major groove of the 20 nt-RBS (e.g. to Thr5_A_), compare (**c**) and (**f**). However, other hydrogen bonds between the SL1 RNA and the protein are formed due to the looping out of the G5 base (f, right). The binding of the two adenines in **A**NGG**A**X [A8 and A12 in 20 nt-RBS, (**b**)] is cooperatively supported by their stacking onto the stem (closing with the C7-G14 base pair), (**a**). In SL1, this stabilization by stacking of the two adenines (A6 and A10 in SL1) onto the stem is not possible, because of the looped-out G5 (**e**). For the 20 nt-RBS, the two *K*_d_ values correspond to the binding of the first and second RNA molecule to the homo-dimeric RsmE protein, respectively.

In SL1, the insertion of two additional nucleotides between the ACGGAU loop and the stem leads to a 10-fold decrease in affinity (compare SL1 with 20 nt-RBS, which have identical loop and stem sequences except for the two inserted nucleotides; Figure 3d). On the one hand, this insertion leads to a lower number of hydrogen bond contacts from the protein side-chains with the functional groups of the bases in the major groove of the stem when the GC-UA closing base pair is shifted down by one register in SL1 compared with the 20 nt-RBS (compare Figure 3c and f). For example, the hydrogen bond of the threonine Thr5_A_ side-chain with the carbonyl O6 of the guanine of the C**G** stem closing base pair is absent in SL1. However, this loss in hydrogen bond contacts between protein and the RNA major groove of SL1 is compensated by new hydrogen bonds of the looped-out G5 base with the side-chains of Arg6_A_ and Glu46_B_ (Figure 3f, right). Although the number of hydrogen bonds between the protein and the RNA cannot explain the affinity difference between SL1 and the 20 nt-RBS RNA for RsmE, the lower affinity of SL1 for RsmE likely arises from the fact that the looping out of the G5 base in SL1 results in a loss of the favourable stacking interactions with the first adenine (A6) of the **A**(N)GGAX motif and with C4 in the stem (Figure 3e), contacts which are present in all other SLs (see stacking of C7 onto A8 and U6 in the 20 nt-RBS, Figure 3a). This stacking interaction might be important for positioning and cooperatively stabilizing the **A**(N)GGAX adenine (A8 in the 20 nt-RBS RNA) at an optimal place for forming strong hydrogen bond interactions with the backbone of the protein (Figure 3b).

### Adaptation of protein side-chains or RNA conformation for recognition of different RNA sequences by the same protein

While the presence of a looped-out nucleotide N in A(N)GGAX or the insertion of two nucleotides between the ANGGAX hexa-loop and the stem influence the binding affinity for RsmE by one to three orders of magnitude, several RNA constructs have comparable affinities despite significant structural differences (low micromolar affinity, Figure 1). SL3 and SL4 have different nucleotides in the upper part of their stems, whereas the 9 nt-GGA_39-41_ RNA is even missing the stem entirely. Comparing the structures of these RNA constructs shows that not only the protein side-chains, but also the RNA can adapt to each other in such a way that various RNA sequences can be bound by RsmE with similar thermodynamic stability.

**Figure 4.**
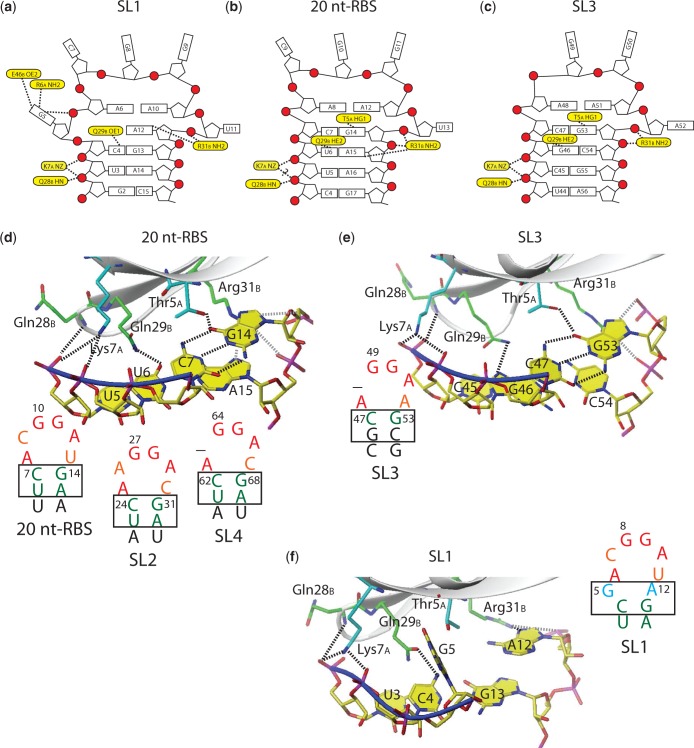
Differential RNA stem recognition. (**a–c**) Schematic representation of intermolecular RNA–protein interactions, yellow residues are involved in potential hydrogen bond contacts. (**d–f**): Structural details of interactions important for recognition. (d) 20 nt-RBS (which shows same contacts as SL2 and SL4), (e) SL3. (f) SL1.

The 5′-nucleotide of the penultimate stem closing base pair is specifically recognized by the glutamine Gln29_B_ side-chain independent of its identity (Figure 4a–c). In SL2, SL4 and 20 nt-RBS, the uracil base of the penultimate stem closing base pair is hydrogen bonded by its O4 carbonyl to the Gln29_B_ HE2 protons (Figure 4d), whereas the same protons are contacting the N7 of the guanine G46 in SL3 (Figure 4e). Although we have not solved a structure containing an adenine at this position, it is likely that an adenine would also be recognized by its N7. The SELEX-derived consensus sequence has selected an adenine at this position (14). In contrast, in SL1 the OE1 carbonyl group of the Gln29_B_ side-chain rather than its HE2 protons contact the amino group of the cytosine C4 (Figure 4f). This nicely demonstrates that the same side-chain can recognize any base through a slight movement or rotation, thereby interacting with a different functional group. Although the glutamine Gln29_B_ side chain recognizes the functional groups of bases, the resulting hydrogen bonds do not contribute to any base discrimination at this position. Similarly, the arginine Arg31_B_ protein side-chain can adapt its conformation to form hydrogen bonds to different RNA bases located in the stem (Figure 4 and Supplementary Text).

**Figure 5.**
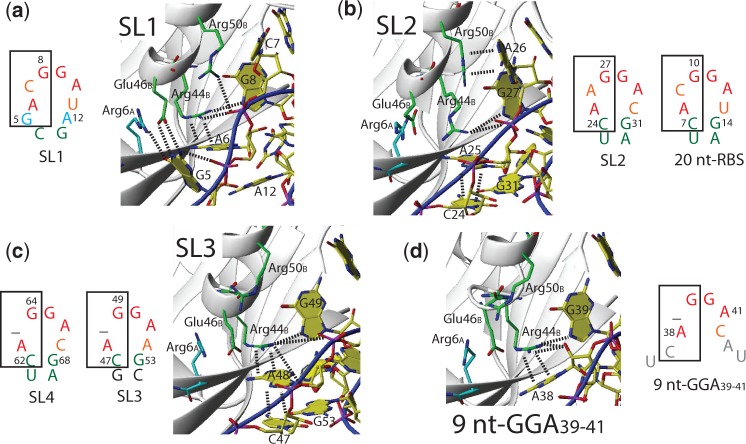
Differential hairpin loop recognition. (**a–d**): Structural details of interactions important for recognition. (a) SL1, (**b**) SL2 (which shows same contacts as 20 nt-RBS). (**c**) SL3 (which shows same contacts as SL4). (d) 9 nt-GGA_39-41_.

Not only can the protein side-chains adapt to recognize different RNA sequences but so can the RNA. In SL2 and 20 nt-RBS, arginine Arg44_B_ contacts the phosphate backbone of the RNA loop but is not involved in stacking interactions with the RNA (Figure 5b). In constrast, in SL1, SL3, SL4 and the 9 nt-GGA_39-41_ RNA, Arg44_B_ stacks on the adenine of the **A**(N)GGAX motif (Figure 5a, c and d). This is possibly due to a slight rearrangement of this adenine base, when either no looped out nucleotide N is present (SL3 and SL4), no stem is present (9 nt-GGA_39-41_ RNA) or two additional nucleotides are inserted between the stem and the A(N)GGAX loop (SL1). Intriguingly, in the single-stranded 9 nt-GGA_39-41_ RNA, the Arg44_B_ side-chain is not only stacking on the adenine A38 of the **A**(N)GGAX motif, but is also forming a hydrogen bond to the A38 2′-hydroxyl group (Figure 5d). This is only possible because the sugar pucker conformation is in C2′-*endo* and not C3′-*endo* like in all stem–loop RNA targets (Figure 5d). Experimental NMR evidence for the C2′-*endo* conformation comes from the 2D ^1^H-^1^H TOCSY spectrum showing strong correlation peaks between H1′ and both H2′ and H3′ (25). In addition, the chemical shifts of the C1′ and C4′ of adenine A38 are clearly indicating a C2′-*endo* conformation (38,39). Overall, the structures of both the RNA and the protein can adapt their structure such that RsmE can bind to different RNA targets with similar affinity.

### GGA motifs within secondary structures bind with mM affinity but show slow exchange with respect to the NMR time scale

**Figure 6.**
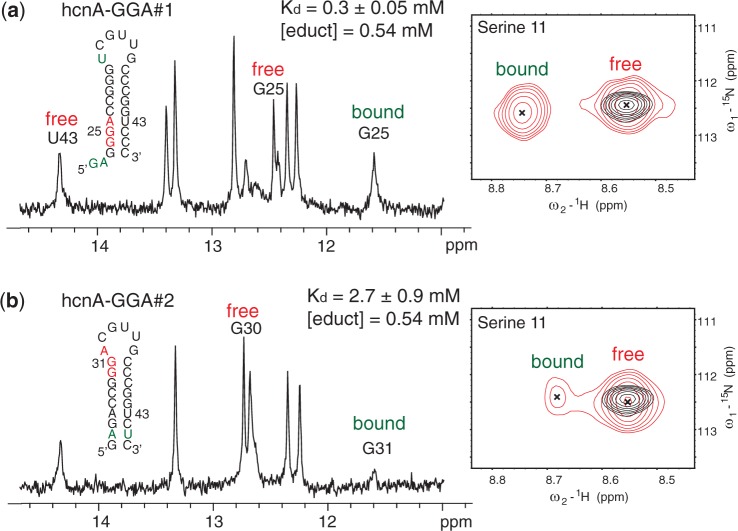
Low affinity GGA motifs are in slow exchange on the NMR chemical shift time scale. (**a** and **b**) Determination of the binding affinity of the two GGA motifs located in SL1 of the 5′-UTR of the *hcnA* mRNA (*K*_d_‘s in the mM range) could be performed by NMR because the resonances are in the slow exchange regime permitting to observe the free RNA, the free protein and the complex at the same time (for details see experimental part). Shown is the imino RNA region of the one-dimensional proton NMR spectrum (left) and the serine 11 amide region of the ^1^H-^15^N HSQC spectrum (right) of the hcnA-GGA#1 (a) or the hcnA-GGA#2 RNA (b) in complex with RsmE. In the ^1^H-^15^N HSQC spectrum, the free RsmE protein (black) is superimposed onto the protein–RNA complex (red). The full spectra are shown in Supplementary Figure S1. The initial educt concentrations were 0.54 mM for the RNA and the protein (monomer concentration). All the spectra were measured at 313 K. The GGA binding motifs are coloured in red, mutations or non-native nucleotides are coloured in green. For the hcnA-GGA#1, a GA dinucleotide was placed at the 5′-end to prevent quadruplex formation observed when omitting the GA. To conserve the secondary structure in the hcnA-GGA#2 RNA, the mutation of the GGA to AGA was compensated with a mutation of C45 to U45 in the opposite strand of the stem.

We determined structures of GGA motifs located either in loops of hairpins or single-stranded regions with affinities for RsmE covering more than two orders of magnitude (∼10–3500 nM). Yet, other GGA motifs are partially or entirely buried within secondary structures and would therefore not be expected to bind the CsrA/RsmE protein. Two such GGA motifs are present in SL1 of the 5′-UTR of the *hcnA* mRNA in *P. fluorescens* (17). As NMR spectroscopy allows detecting weak interactions, we individually titrated two mutant RNA constructs containing either of the two GGA motifs into RsmE. We detected resonances with chemical shifts characteristic of complex formation (Figure 6 and Supplementary Figure S1). Strikingly, however, by titrating two equivalents of the mutant RNAs into one equivalent of RsmE protein homo-dimer, we could simultaneously observe the free and the bound protein amide resonances and notably also the free and bound RNA imino resonances (Figure 6). This shows that first, the dissociation constants of theses complexes are in the range of the protein and RNA concentrations used during the NMR chemical shift titration (∼0.5 mM) and second, that the complexes are in the slow exchange regime compared with the NMR chemical shift time scale. This finding is unexpected because low-affinity protein–RNA complexes are usually found to be in the fast exchange regime due to a high rate of complex association and dissociation [k_on_ in the order of 10^6^–10^8^/Ms (25,40)]. Thus, a low-affinity complex being in slow exchange on the NMR chemical shift time scale implies a small apparent association rate (41). A small apparent association rate can be rationalized by the following two scenarios: the RsmE protein can only bind to a single-stranded GGA motif (presented as single-stranded region or hairpin loop). Thus, binding of RsmE to a GGA motif partially or entirely buried within a secondary structure can only occur during the short time window in which the secondary structure is transiently opened and thus, is present in an accessible, binding competent state (conformational selection). This decreases the fraction of productive complex formation events and thereby the apparent association rate. The second possibility is that RsmE binds non-specifically to the buried GGA-binding motif, which is followed by a slow structural rearrangement (induced fit) forming the cognate protein–RNA complex, again having a small apparent association rate as a consequence.

In summary, we demonstrate that not only the sequence surrounding a certain GGA motif contributes to the modulation of the binding affinity for RsmE but that the secondary structural context influences the range of binding affinities of the Csr/RsmE protein–RNA recognition system to extend over five orders of magnitude.

### Framework for predicting binding affinity of GGA motifs for CsrA/RsmE

**Figure 7.**
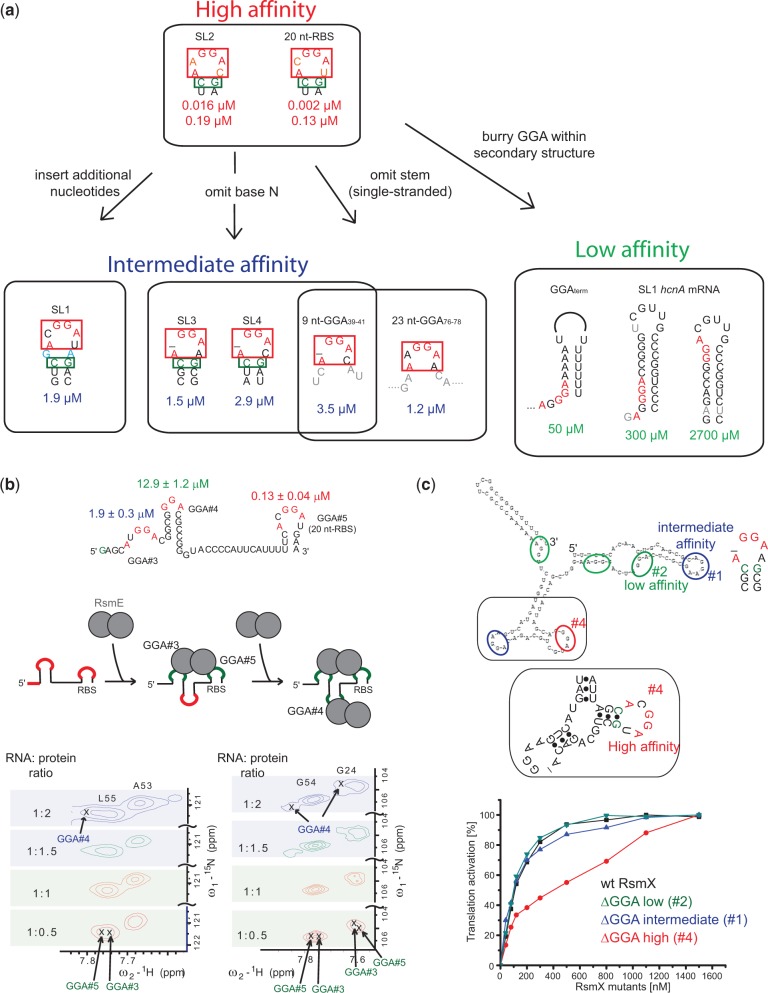
Framework for predicting the binding affinity of a certain GGA motif for the CsrA/RsmE type protein. (**a**) Overview of the Csr/Rsm protein–RNA recognition. For the high affinity binding targets, the ITC titration curves could only be fitting using a two-site binding model. All *K*_d_ values were determined by ITC, except the ones of SL1 of the *hcnA* mRNA, which were determined by NMR. (**b**) Binding of RsmE to the 5′UTR of the *hcnA* mRNA. Shown is the secondary structure of a truncated version of the 5′-UTR of the *hcnA* mRNA containing GGA#3-#5. A zoom into two representative regions of ^1^H-^15^N HSQCs recorded at different ratios of protein and RNA (0.5, 1, 1.5, 2) are presented. Although the resonances characteristic for GGA#3 and GGA#5 are present throughout the titration, peak characteristic for GGA#4 start to appear when more than one equivalent of RsmE dimer is added to the RNA. (**c**) *In vitro* translation assay assessing the translation activation potential of RsmX sRNA wild-type and mutants in which various GGA motifs were mutated to abolish their binding (GGA → AGA mutations).

Our study allows the proposal of a high affinity binding consensus sequence, which is defined by a hexa-loop of the form **A**N**GGA**X placed directly on top of a stem (Figure 7a). Modifying this high affinity binding motif leads to a decrease in binding affinity. The occlusion of the GGA motif within a base paired region has the largest impact in reducing the binding affinity. Smaller variations in binding affinity (10 to 1000-fold) can be achieved by omitting the nucleotide N in A(N)GGAX (SL3 and SL4), inserting additional nucleotides between the ANGGAX hexa-loop and the stem (SL1) or entirely lacking a stem (23 nt-GGA_76-78_ and 9 nt-GGA_39-41_, Figure 7a).

Having a framework for predicting the affinity of a particular GGA motif for the CsrA/RsmE protein, we asked ourselves if we could predict the binding of RsmE to other mRNAs and sRNAs eventually making predictions of the effect of certain sRNA or mRNA mutations on translation activation or repression, respectively.

First, we aimed to predict the binding of RsmE to the 5′-UTR of the *hcnA* mRNA from *P. fluorescens* (Figure 7b). Aside from two GGA motifs binding with low mM affinity (Figure 6), the 5′-UTR contains three additional GGA motifs. On the basis of the GGA motif sequences, we expect that the GGA#3, #4 and #5 motifs in isolation bind RsmE with intermediate, intermediate–low and with high affinity, respectively. These predictions were verified by ITC (Figure 7b and Supplementary Figure S2). We performed NMR chemical shift titrations to observe the binding of ^15^N-labelled RsmE to an unlabelled minimal fragment of the *hcnA* mRNA 5′-UTR containing the three GGA#3–#5 motifs. Unsurprisingly, the first RsmE protein dimer binds simultaneously to GGA#3 and GGA#5, whereas a second RsmE dimer binds to GGA#4, which is the weakest binding motif. These findings suggest that one RsmE dimer binding to both GGA#3 and GGA#5, which overlaps with the RBS, is responsible for translation repression of the *hcnA* mRNA. These observations are in agreement with previous *in vivo* results demonstrating that mutations in GGA#3 and #5 significantly reduced translation repression by RsmE, while mutations in GGA#4 only slightly affected repression (11,17).

Next, we investigated the effect of single point mutations in different GGA motifs of the sRNA RsmX on translation activation of the *hcnA* mRNA of *P. fluorescens* (Figure 7c). The RsmX sRNA in *P. fluorescens* contains six GGA motifs which are predicted to include one high, two intermediate and three low affinity sites for RsmE. By positioning the 5′-UTR of *hcnA* mRNA upstream of the reporter gene CAT, we tested by cell-free expression the activation potential of wild-type RsmX compared with three RsmX mutants in which either the predicted high, intermediate or low affinity GGA motif was mutated in order to abolish its binding. Notably, mutating the site predicted to bind with high affinity strongly reduced translation activation, while the predicted intermediate and low affinity site mutants had only a slight or no effect on translation activation, respectively. These observations establish that strong CsrA/RsmE binding sites on sRNAs more efficiently sequester the RsmE protein from the RBS of mRNAs and are therefore more competent in activating translation initiation.

In conclusion, these two examples show that our framework for correlating a certain GGA motif to its binding affinity for CsrA/RsmE can be used to successfully predict the function/effect of certain GGA motifs/mutations on translation activation or repression.

## DISCUSSION

We have elucidated and compared the solution complex structures of RsmE bound to six different target RNAs, which are the four SLs and the single-stranded region between SL2 and SL3 of the sRNA RsmZ (this study) and a 20 nt RNA SL encompassing the RBS of the *hcnA* mRNA in *P. fluorescens* (11). All RNA target sequences contain a common **A**(N)**GGA**X binding motif but the context of the sequence modulates the binding affinity for RsmE by more than two orders of magnitude. When the GGA motif lies partially or entirely within a base paired region, the affinity can further decrease by two to three orders of magnitude, resulting in a total modulation of the binding affinity of the Csr/Rsm system to more than five orders of magnitude. The degree of binding affinity attenuation is given by the extent by which the RNA secondary structure has to be disrupted in order to bind RsmE (compare in Figure 7 the three low affinity binding constructs). Remarkably, all the tested GGA binding motifs are in slow exchange in respect to the NMR chemical shift time scale independent of their binding affinity. This can be explained in that the rate of complex dissociation is slow (<10/s) for all complexes of this family of protein–RNA interactions and that the binding affinity covering five orders of magnitude is significantly affected by the variable rate of complex association. The slow rate of complex dissociation is provided by the conserved **A**(N)**GGA**X binding motif, which is recognized by many hydrogen bonds to the backbone of the RsmE protein. As this recognition involves binding of the nucleobases by the backbone and not the side-chains of the protein, this recognition is specific and these bases cannot be replaced by other ones and still be accommodated by the given protein fold. In contrast to the core **A**(N)**GGA**X motif, the nucleotides N and X and the nucleotides adjacent to the core motif are variable and allow for a modulation of the binding affinity.

We present a toolkit for predicting the binding affinity of different GGA motifs for the Csr/Rsm proteins (Figure 7a). While a ANGGAX hexa-loop directly placed on top of a stem contributes to high affinity binding, omitting the nucleotide N in A(N)GGAX (SL3 and SL4), inserting additional nucleotides between the ANGGAX hexa-loop and the stem (SL1), removing the entire stem (GGA_76-78_ and GGA_39-41_) or more severely, occluding GGA motifs within secondary structures lead to lower binding affinity. Interestingly, AGGGA and AAGGA pentaloop sequences have very recently been shown to have high affinity for RsmE, thus suggesting that omitting the nucleotide X in A(N)GGAX when the nucleotide N is a purine also constitutes a high affinity binding target for CsrA/RsmE (42).

Unexpectedly, omitting a stem when N in A(N)GGAX is missing does not change the binding affinity considerably. The single-stranded 9 nt-GGA_39-41_ RNA has a similar affinity like SL4, although the 9 nt-GGA_39-41_ RNA contains an identical loop sequence but does not have a stem (Figure 7a) and therefore lacks all the hydrogen bond contacts with the RsmE protein provided by the stem. Furthermore, it is expected that the binding of a single-stranded RNA is entropically disfavoured compared with binding of a pre-formed stem–loop RNA. Yet, this discrepancy can be explained at least partially by the additional H-bond from Arg44 to the A38 2′-hydroxyl group which is cooperatively enforced by the Arg44/A38 stacking interaction. The importance of this interaction is supported by a recent finding that a single-stranded DNA (ssDNA) (missing the 2′-hydroxyl group) containing a GGA motif did not bind the homologous CsrA protein, whereas a GGA motif located in the loop of a hairpin DNA did (43). Interestingly, this additional H-bond can only form because of a structural adaptation of the RNA. The adenine A38 of the **A**(N)GGAX in the single-stranded 9 nt-GGA_39-41_ RNA has a C2′-*endo* sugar pucker conformation, while an equivalent adenine stacking on a stem has a sugar pucker C3′-*endo* conformation. RNA adaptation leading to recognition of different RNA sequences with equal affinity has been demonstrated recently. The solution structures of the oligonucleotides 5′-UCCAGU-3′ and 5′-UGGAGU-3′ in complex with the RRM domain of SRSF2 revealed that the cytosines having an ‘anti’-base conformation are recognized very similarly as the guanines having a *syn* conformation (44). One arginine side-chain recognizes either the Watson–Crick edge of the cytosine or the Hoogsteen edge of the guanine.

Interestingly, our structures reveal that also adaptation of the protein side-chains allows the recognition of different RNA sequences. For example, Gln29 can recognize all four bases by a simple adjustment of its side-chain position and orientation. Alternative side-chain conformations have also been suggested to contribute to the degenerate recognition of polypyrimidine tracts by U2AF^65^ (45), the recognition of multiple RNA targets by the specific fem-3 binding factor (FBF) protein of the PUMILIO/FBF (PUF) family (46) or three different RNA targets by Lin28 (47). Very recently, the X-ray crystal structures of Pot1pC in complex with its cognate and several non-cognate ssDNA ligands have nicely demonstrated that changes in nucleic acid and protein structure (such as rotation of bases or protein side-chains) allows for alternative H-bond networks or stacking interactions and thus for the accommodation of several different DNA sequences by the same protein with thermodynamic equivalence (48). Thus, adaptation of protein and nucleic acid seems to be a general mechanism contributing to the binding affinity in protein-nucleic acid recognition, without conferring specificity, supporting recent findings that non-specific and specific RNA-binding modes may not differ fundamentally (49).

Notably, despite their distinct protein folds, the orthologues RsmE protein from *P. fluorescens* (this study) and the RsmN protein from *P. aeruginosa* (12) bind SL2 from the corresponding RsmZ sRNA in an almost identical fashion (Supplementary Figure S6). Simply, the looped-out adenine (corresponding to nucleotide N of the A(**N**)GGAX motif) has a different structure. This is due to the α-helix, which is only present in RsmE but not in RsmN.

In contrast to the CsrA/RsmE system, in which the protein binding surface is almost identical in all the homologous proteins and the variety of RNA sequences are recognized by the same protein by adaptation of protein side-chain and RNA conformations, the coat proteins of single-stranded bacteriophages recognize their cognate RNA targets by very distinct binding modes. The co-evolution of coat protein and corresponding RNA structure ensures that each coat protein specifically binds only its cognate RNA target and discriminates against other RNA targets (50,51).

Fine-tuning of the binding affinity for CsrA/RsmE over more than five orders of magnitude allows RNA (mRNA or the competing sRNA) to adjust its affinity according to its specific function in the cell. Different affinities of mRNAs for CsrA/RsmE allow for a fine-tuning of translation repression if CsrA/RsmE binds to the RBS or close to it. Strong binding of CsrA/RsmE to the RBS increases competition with the 30S ribosomal subunit for binding to the RBS, hence stronger translation repression (Figure 7b) (11,17). Likewise, strong CsrA/RsmE binding sites on a sRNA result in a more efficient translation de-repression by an increased sequestration capability of the sRNA (Figure 7c). Furthermore, different binding affinities for CsrA/RsmE could also modulate the lifetime of mRNAs or sRNAs, which are stabilized from degradation upon CsrA/RsmE binding. The CsrA protein has been shown to increase the lifetime of the *flhDC* mRNA by binding to its 5′-UTR (52,53) and mutations of three or five out of the seven GGA motifs in the sRNA RsmY in *P. fluorescens* have lowered its stability significantly (20). We speculate that strong binding of CsrA/RsmE to a potential RNase E cleavage site would protect the RNA more efficiently against endonucleolytic attack because CsrA/RsmE would better compete with RNase E for the binding/cleavage site.

Considering the estimated total concentrations of the CsrA protein in the range of 6–17 µM in *E. coli* (54), it is legitimate that the GGA motif overlapping the RBS (nM affinity) is binding RsmE under physiological conditions (11,17). In contrast, the two GGA motifs located partially within the stem of SL1 of the *hcnA* mRNA are not assumed to be relevant for binding RsmE *in vivo* (dissociation constants of 300 µM and 2.7 mM). It has been shown that chromosomally transcribed mRNAs are not homogenously expressed in the cellular space of *E. coli* (55). Thus, locally, the concentration of CsrA might largely exceed concentrations in the low to intermediate µM range and binding might also be relevant with *K*_d_ values in the low mM range. Interestingly, mutations disrupting these GGA motifs had an impact on *hcnA* gene expression (17). It is possible that these mutations simply destabilize SL1 leading to a reduced stability of the *hcnA* mRNA (56). Another possibility is that modulation of the secondary structure of the *hcnA* mRNA could lead to an exposure of the buried GGA motifs and thus significantly increase their affinity for the CsrA/RsmE protein. *In vivo*, the secondary structure (or even the tertiary structure) of an mRNA could be modulated by several factors that could vary in a cell state-specific manner. It is credible that sRNAs base pair with the 5′-UTR of the mRNA as has been proposed for RsmY and the *hcnA* mRNA 5′-UTR in *P. fluorescens* (20). In addition, it is conceivable that protein factors such as Hfq (57), small molecules (58), ions (e.g. Mg^2+^) (59), the temperature (60) or co-transcriptional folding (61) could modulate the accessibility of buried GGA motifs by stabilization of alternative secondary structures. Specifically, the possibility that an mRNA is transcribed in the presence of CsrA/RsmE could allow the binding of the protein to a GGA motif for which the complementary secondary structural element has not been transcribed yet. A rearrangement of the entire secondary structure and the binding of otherwise inaccessible GGA binding motifs could be the consequence.

In conclusion, this work exemplifies the enormous diversity in the Csr/Rsm protein–RNA recognition and provides the basis for the next level of binding complexity, which is the assembly of several CsrA/RsmE homo-dimeric proteins on a sRNA or mRNA containing more than one GGA motif on a single molecule.

## ACCESSION NUMBERS

Atomic coordinates, NMR chemical shifts and NMR restraints for the structures of the SL1/RsmE, SL2/RsmE, SL3/RsmE, SL4/RsmE and 9 nt-GGA_39-41_/RsmE complexes have been deposited in the Protein Data Bank under accession codes 2mfc, 2mfe, 2mff, 2mfg and 2mfh, respectively.

## SUPPLEMENTARY DATA

Supplementary Data are available at NAR Online.

## FUNDING

The Swiss National Science Foundation [3100A0-118118, 31003ab-133134 and 31003A-149921 to F.H.-T.A]; the SNF-NCCR structural biology Iso-lab. Funding for open access charge: NAR Editorial Board members are entitled to one free paper per year in recognition of their work on behalf of the journal.

*Conflict of interest statement*. None declared.

## Supplementary Material

Supplementary Data
