# Supplementary material for: Molecular basis for the wide range of affinity found in Csr/Rsm protein–RNA recognition
Source: Nucleic Acids Res. 2014 Feb 21;42(8):5332–46. doi: 10.1093/nar/gku141 (PMC4005645; doi:10.1093/nar/gku141)
Supplement: Supplementary Data [file supp_42_8_5332__index.html]

Molecular basis for the wide range of affinity found in Csr/Rsm protein–RNA recognition — Molecular basis for the wide range of affinity found in Csr/Rsm protein–RNA recognition — Supplementary Data 

# Molecular basis for the wide range of affinity found in Csr/Rsm protein–RNA recognition

## Supplementary Data

files

**Files in this Data Supplement:**

- Supplementary Data - pdf file
