# Supplementary material for: Molecular basis for the wide range of affinity found in Csr/Rsm protein–RNA recognition
Source: Nucleic Acids Res. 2014 Feb 21;42(8):5332–46. doi: 10.1093/nar/gku141 (PMC4005645; doi:10.1093/nar/gku141)
Supplement: Supplementary Data [file supp_gku141_nar-03723-r-2013-File009.pdf]

Supplementary information

Molecular basis for the wide-range of affinity found in Csr/Rsm protein-RNA recognition

Olivier Duss, Erich Michel, Nana Diarra dit Konté, Mario Schubert and Frédéric H.-T. Allain*

Supplementary Text

Interactions of the common A(N)GGAX binding motif

The two guanine bases of the common **A**(N)**GGA**X motif are specifically recognized by hydrogen bonds from the backbone atoms in the $\beta5_B$-strand and the preceding $\beta4_B$-$\beta5_B$ loop ($Pro37_B$, $Val40_B$, $Val42_B$ and $Arg44_B$) and are packed against the hydrophobic side-chains of $Leu2_A$, $Leu4_A$ and $Val42_B$ (Supplementary Figure S4a, b and reference (11)). The two adenines are coplanar but do not interact with each other and make specific hydrogen bonds via their Watson-Crick (the 5′-adenine) or their Hoogsteen (the 3′-adenine) edges to the backbone of $Thr5_A$ or $Ile3_A$, respectively (see Supplementary Figure S4a, d). In addition, the base and sugar of the 3′-adenine (A29 in SL2) pack against the hydrophobic side-chain of isoleucine $Ile3_A$. The looped-out nucleotide X is not specifically recognized but makes hydrophobic contacts to the side-chains of $Met1_A$ and $Leu23_B$ (Supplementary Figure S4c). The NH_3^+ of the N-terminal methionine as well as the positively charged side chain of lysine $Lys38_B$ stabilize the phosphate backbone of the RNA by salt bridges (Supplementary Figure S4c).

Differential RNA recognition by the arginine Arg31 side-chain

In those SL's containing CG-UA closing base-pairs (SL2, SL4 and 20nts-RBS), the side-chain of arginine Arg31 specifically recognizes the N7 of the adenine of the penultimate stem closing base-pair (CG-U**A**) but not the N7 of the guanine base of the ultimate stem closing base-pair (C**G**-UA) (see e.g. A15 in 20nts-RBS in Figure 4d). In SL1, the two additional inserted nucleotides G5 and A12 shift the CG-AU closing base-pair downwards by one base-pair, such that in SL1 the guanine base G13 of the ultimate C**G**-UA closing base-pair is placed at the equivalent position as the adenine A15 of the CG-U**A** penultimate stem closing base-pair in 20nts-RBS (compare the secondary structures of SL1 and 20nts-RBS in Figure 4). It would be expected that the Arg31 side-chain (which recognizes the N7 of the adenine A15 in 20nts-RBS, Figure 4d) recognizes the N7 of the guanine G13 in SL1. However, in SL1, the Arg31 side-chain makes a specific hydrogen bond to the N7 of the inserted adenine A12 (Figure 4f). This adenine A12 stacks on the C4-G13 closing base-pair but is not base-paired to any nucleotide and therefore adopts a slightly different position than the equivalent G14 of the stem closing base-pair in 20nts-RBS (compare Figure 4d and f). This allows the side chain of Arg31 to make a specific hydrogen bond to the non-base-paired A12 of SL1 but not to the equivalent G14 in 20nts-RBS. The re-positioning of the Arg31 side-chain in the SL1/RsmE complex allows the formation of salt-bridges to the phosphate of A12 but not to the phosphate of G13 in SL1 (Figure 4f). In contrast, the Arg31 side chain can contact both phosphates of G14 and A15 in 20nts-RBS (Figure 4d).

In SL3, the penultimate stem closing base-pair is not a U-A but a G-C (Figure 4e). Whereas in 20nts-RBS (or SL2 and SL4) the adenine A69 is recognized by its N7 by the side-chain of Arg31 (Figure 4d), the cytosine C54 of SL3 at the equivalent position cannot form a hydrogen bond with the Arg31 side-chain (Figure 4e). It is expected that a guanine is as well tolerated as an adenine at this position. It can also be anticipated that a uracil base at the same position would be recognized by its O4 carbonyl acceptor group. Interestingly, the SELEX derived consensus sequence selects a uridine at this position (14). In conclusion, we suggest that any base except for a cytosine is tolerated at this position (3′-base of the penultimate stem closing base-pair).

Supplementary Figures

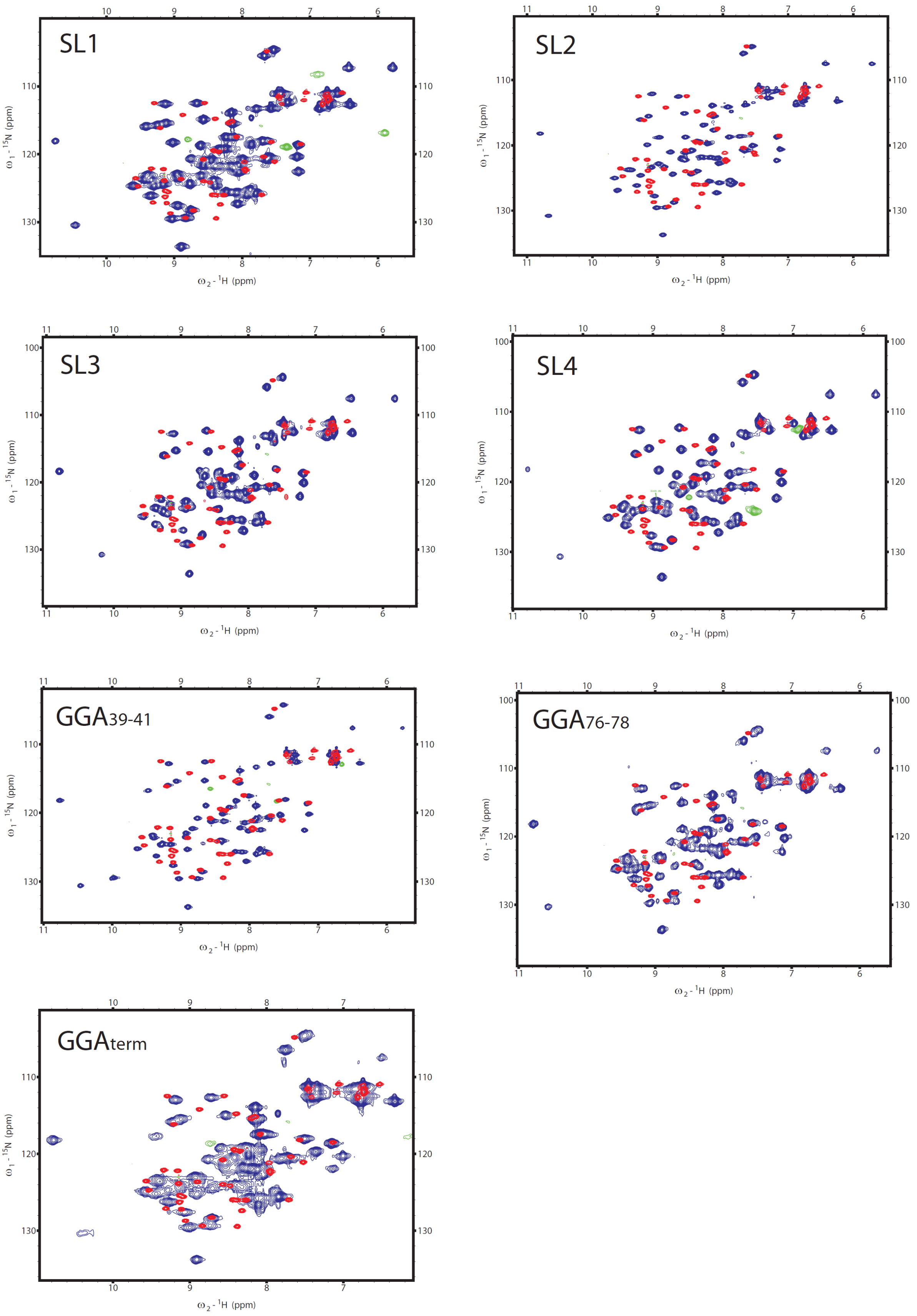

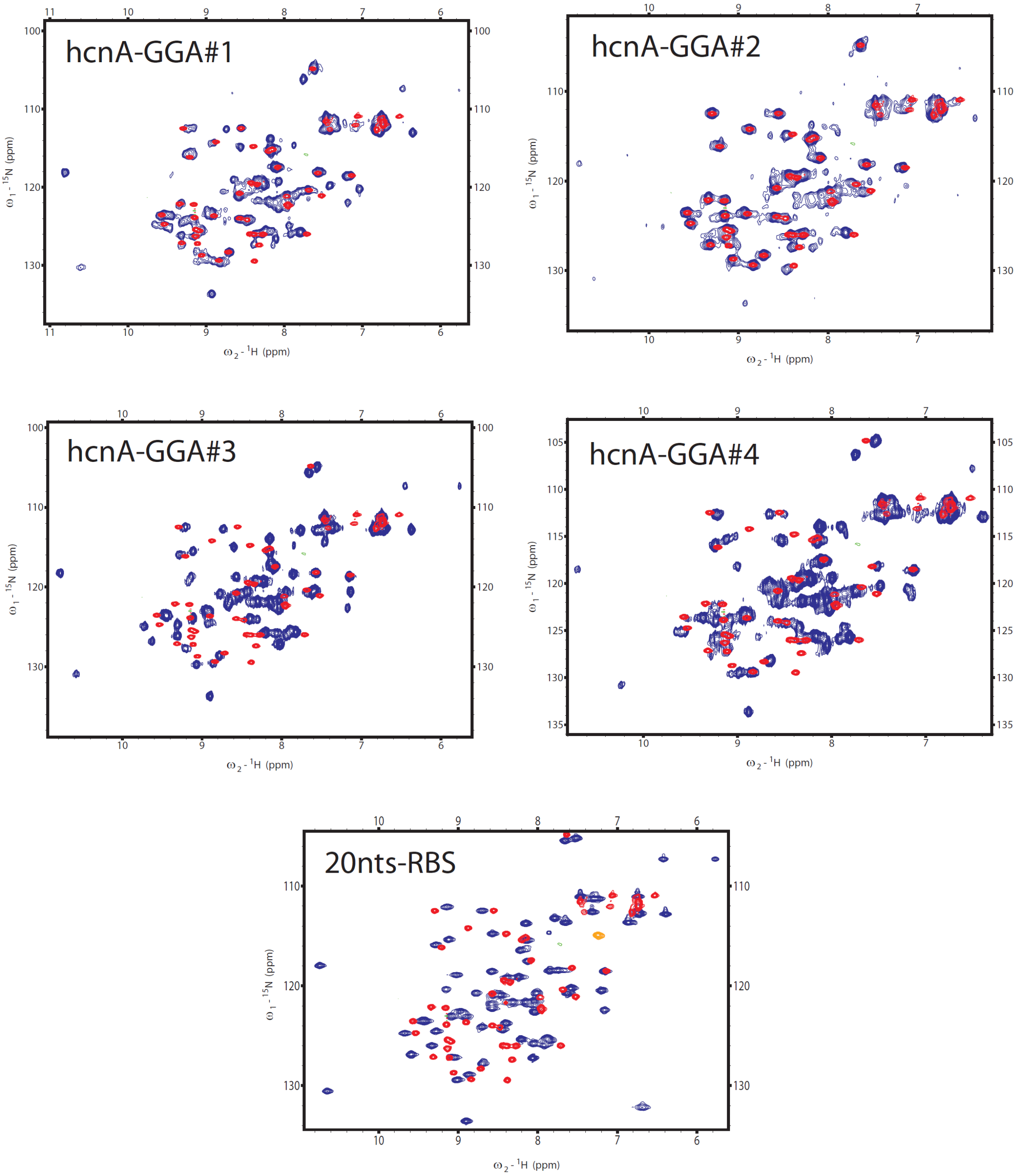

Figure S1: Overview of the ^{1}H-^{15}N HSQC spectra of the complexes used in this study. Shown are the ^{1}H-^{15}N HSQC spectra of the free (red) and bound (blue) RsmE protein. All the spectra were measured at 313 K. The RNA target of the bound RsmE protein is shown on the top left corner of the spectra. The RNA sequences of the RNA targets are presented in Supplementary Table S2.

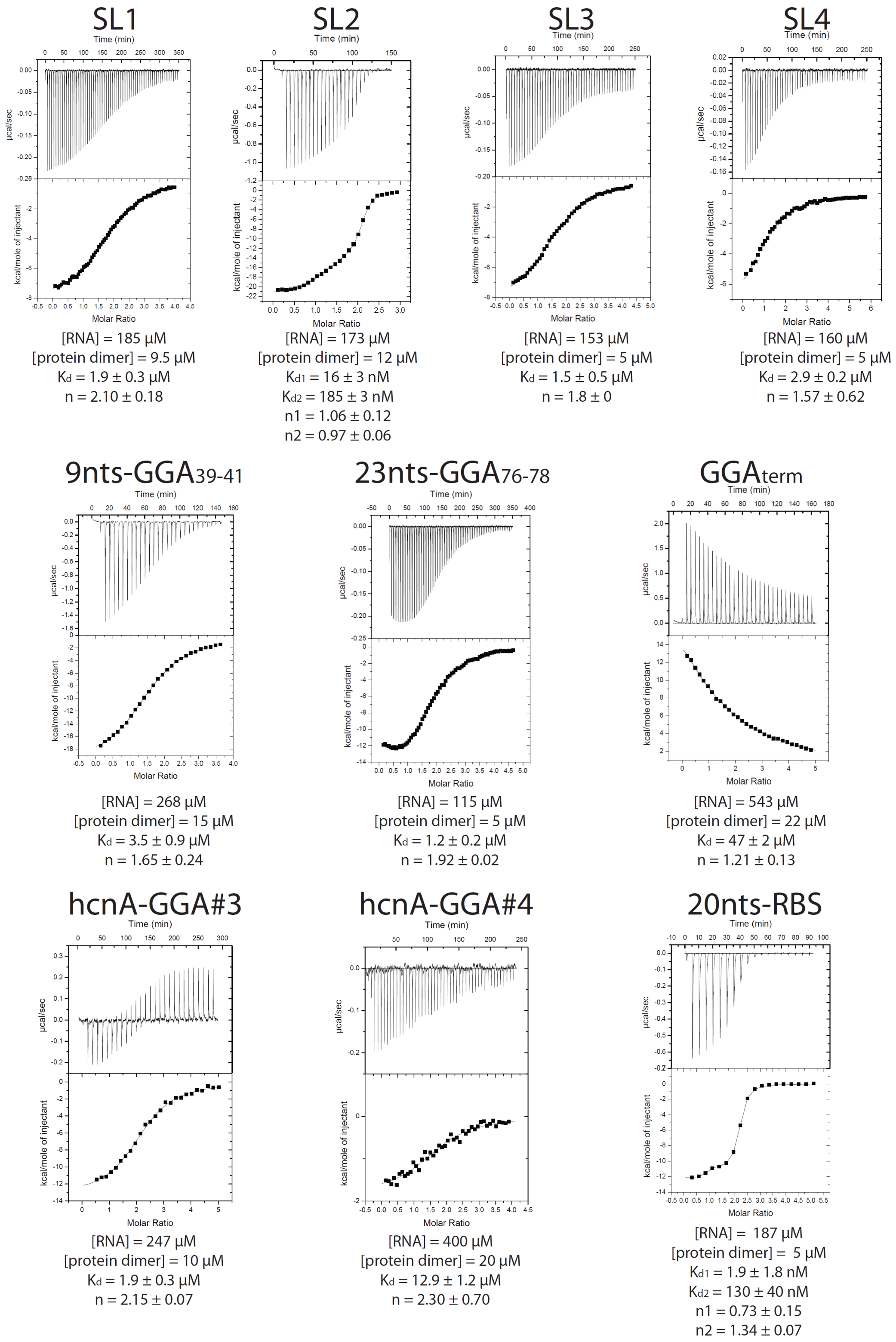

Figure S2: Overview of a representative ITC binding curve of each construct used in this study. The concentrations of the RNA and the RsmE protein homo-dimer (two binding sites) are indicated. The higher concentrated component (in the syringe) was titrated into the lower concentrated component (cell). All measurements were performed at 298 K in 300 mM NaCl and 50 mM potassium-phosphate at pH 8.0. The errors were calculated from at least two independent measurements (standard deviations indicated). SL2 and 20nts-RBS were fitted using a 2-site binding model while all the other binding curves were fitted using a 1-site binding model. The sequences of all the RNA constructs are presented in Supplementary Table S2.

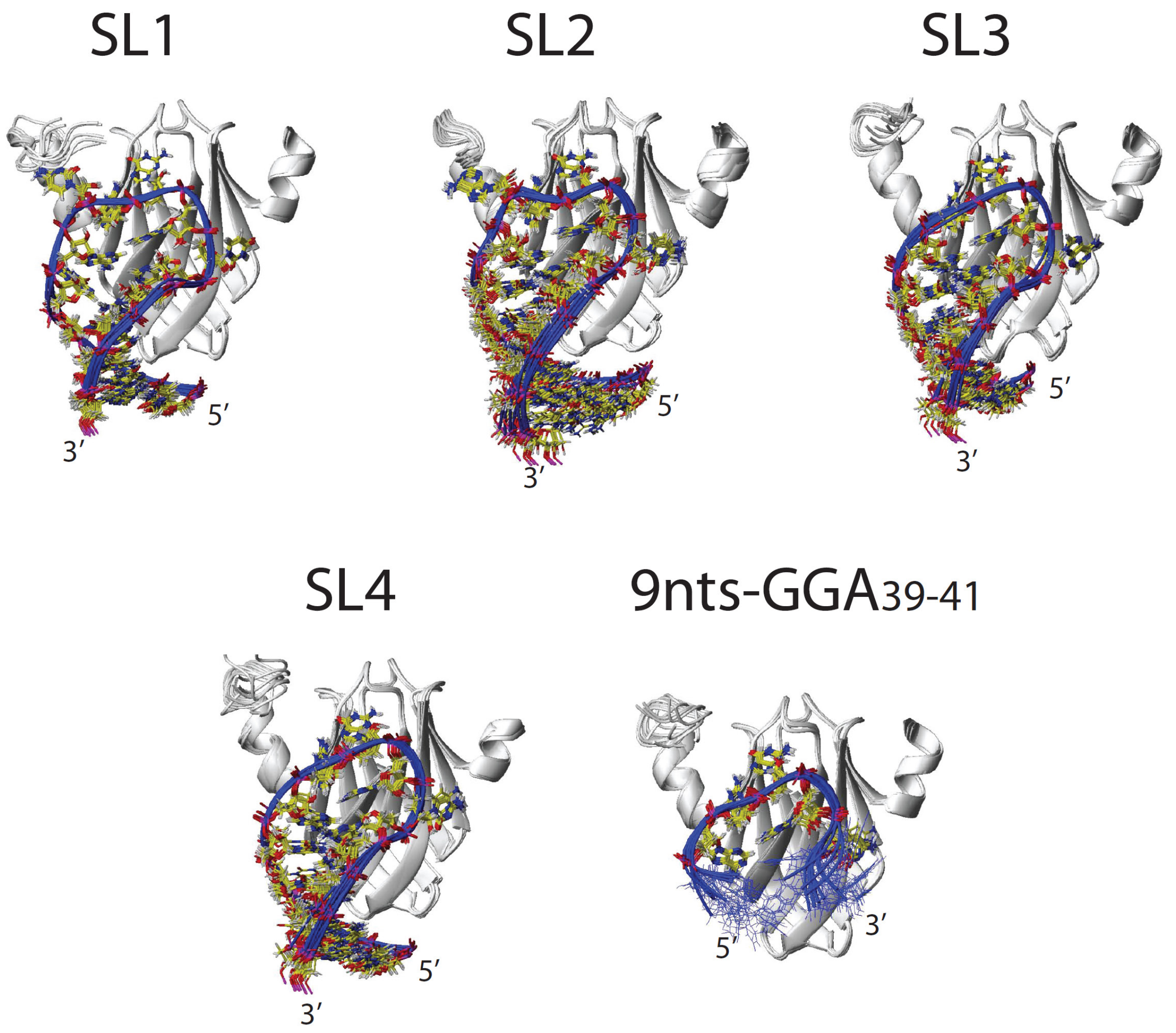

Figure S3: NMR structural ensembles of all 5 complex structures. The second RNA molecule binding to the homo-dimeric RsmE protein is not shown for simplicity. For the 9nts-GGA$_{39\text{-}41}$ RNA in complex, the nucleotides U36, C37, A43 and U44 are unstructured and shown in blue line representation.

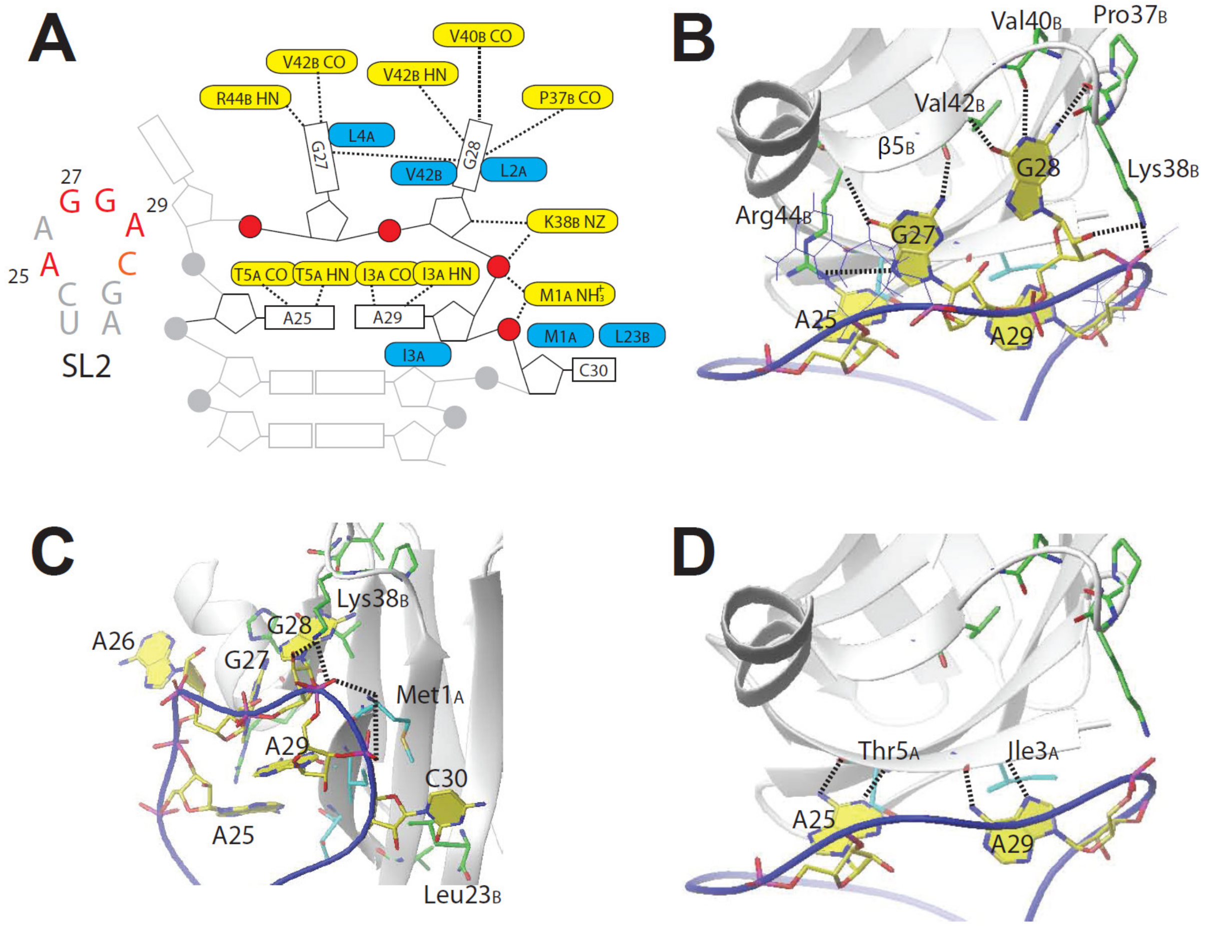

Figure S4: The common binding mode of the **A**(N)**GGAX** motif demonstrated by SL2. (**a**) Schematic representation of intermolecular RNA-protein interactions, blue residues are involved in hydrophobic/stacking interactions, yellow residues in potential hydrogen bond contacts. (**b-d**): Structural details of interactions important for recognition. The nucleotide N (A26 in SL2) is only shown in thin line representation in (**b**) for simplicity.

A

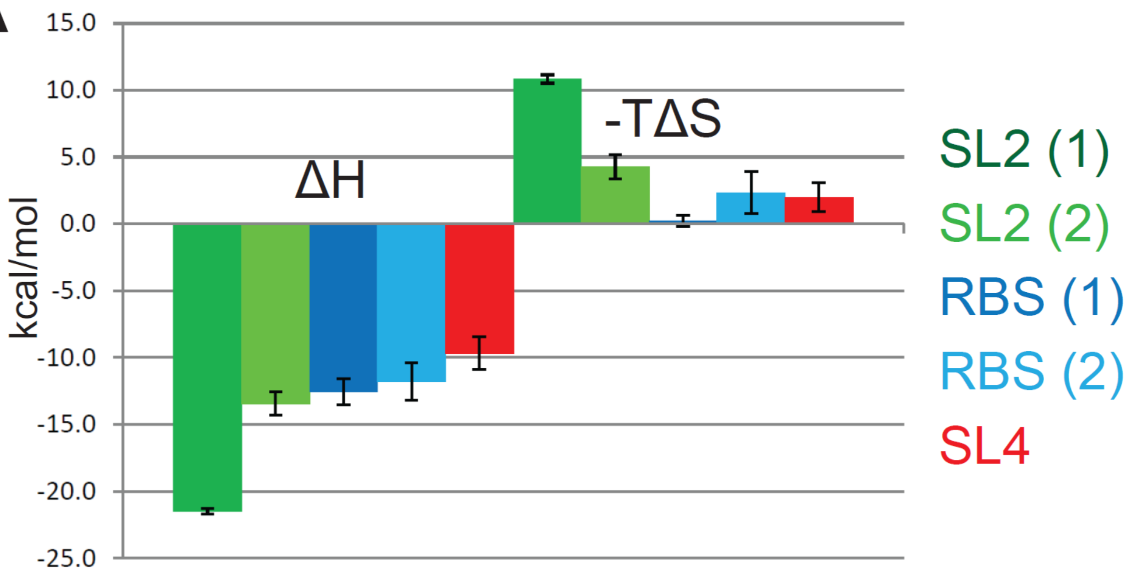

B

	$K_d^{(1)}$	$\Delta H^{(1)}$	$-T\Delta S^{(1)}$	$K_d^{(2)}$	$\Delta H^{(2)}$	$-T\Delta S^{(2)}$
	[nM]	[kcal/mol]	[kcal/mol]	[nM]	[kcal/mol]	[kcal/mol]
SL2	16 ± 3	-21.5 ± 0.2	10.8 ± 0.3	185 ± 3	-13.4 ± 0.9	4.3 ± 0.9
20nts-RBS	1.9 ± 1.8	-12.5 ± 1.0	0.2 ± 0.4	130 ± 40	-11.8 ± 1.4	2.3 ± 1.6
SL4	2900 ± 200	-9.7 ± 1.2	2.0 ± 1.1	-	-	-

Figure S5: Thermodynamic enthalpy/entropy compensation for SL2, SL4 and the 20nts-RBS RNA binding to RsmE. (**a**) Bar representation of thermodynamic changes for SL2, SL4 and the 20nts-RBS RNA. (**b**) Overview of binding affinity, enthalpy and entropy for SL2, SL4 and the 20nts-RBS RNA binding to RsmE. All measurements were performed at 298 K in 300 mM NaCl and 50 mM potassium-phosphate at pH 8.0. The errors were calculated from at least two independent measurements (standard deviations indicated). Note that SL4 has been fitted with a 1-site binding model, while SL2 and 20nts-RBS could only be fitted with a 2-site binding model.

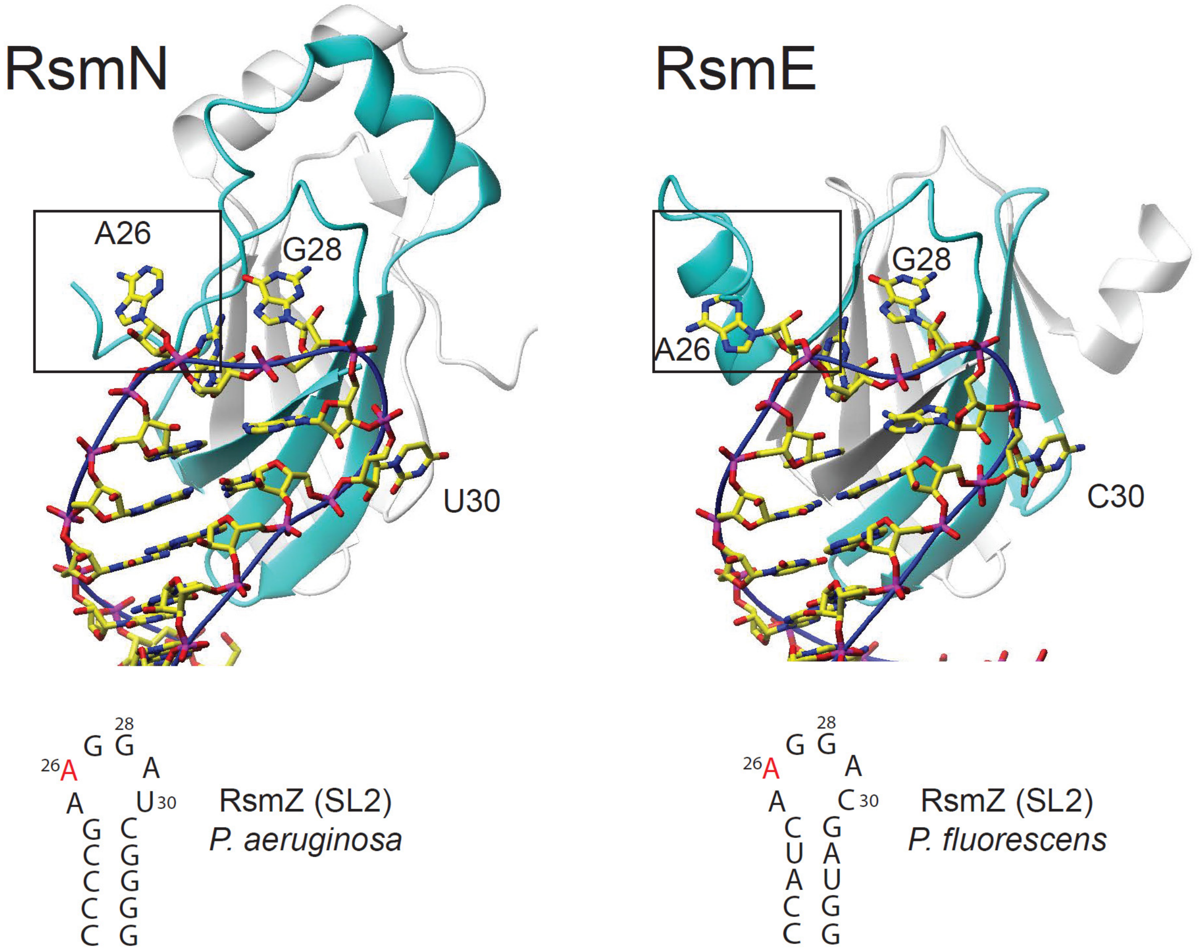

Figure S6: Comparison of the binding mode of the orthologous RsmN and RsmE proteins. Despite the distinct protein folds, the binding of SL2 of the RsmZ sRNA from *Pseudomonas aeruginosa* and *P. fluorescens*, respectively, is almost identical. The only difference is the recognition of the looped-out nucleotide N of the A(**N**)GGAX motif. This difference can be explained by the missing α-helix at the C-terminus in RsmN (see boxed adenine 26).

Supplementary Tables

NMR distance and dihedral constraints (per protein-RNA subunit)	SL1		SL2		SL3		SL4		9nts-GGA$_{38\text{-}41}$	
	Protein	RNA	Protein	RNA	Protein	RNA	Protein	RNA	Protein	RNA
Distance restraints										
Total NOEs (intramolecular)	820	321	780	259	741	249	770	294	776	132
Intra-residue	60	122	147	123	49	96	65	125	65	59
Inter-residue	760	199	633	136	692	153	705	169	711	73
Sequential (|*i*-*j*| = 1)	369	158	326	102	347	113	345	129	352	69
Nonsequential (|*i*-*j*| > 1)	391	41	307	34	345	40	360	40	359	4
Hydrogen bonds (intramolecular)	16	19	17	21	16	22	16	21	16	0
Protein-protein intermolecular NOEs	187		127		139		181		183	
Protein-protein intermolecular hydrogen bonds	10		10		10		10		9	
Protein-RNA intermolecular NOEs	267		184		128		182		154	
Protein-RNA intermolecular hydrogen bonds	8		8		7		7		7	
Total dihedral angle restraints	97	82	101	92	97	91	92	91	92	7
Sugar pucker*		8		6		5		5		7
Backbone†	97	74	101	86	97	86	92	86	92	0

Structure statistics	SL1	SL2	SL3	SL4	9nts-GGA$_{38\text{-}41}$
Violations (mean ± s.d.)					
Number of distance restraint violations > 0.2 Å	0.10 ± 0.32	2.1 ± 0.57	2.3 ± 1.06	1.6 ± 0.84	2.8 ± 1.69
Max. distance constraint violation (Å)	0.16 ± 0.02	0.42 ± 0.02	0.22 ± 0.02	0.27 ± 0.03	0.31 ± 0.10
Number of dihedral angle violations > 5˚	2.8 ± 0.9	3.8 ± 1.1	1.5 ± 1.3	2.6 ± 1.0	3.6 ± 2.5
Max. dihedral angel violation (˚)	16.4 ± 1.8	13.7 ± 5.9	10.2 ± 5.0	14.7 ± 14.2	17.1 ± 15.5
Deviations from idealized geometry					
Bond length (Å)	0.01	0.01	0.01	0.01	0.009
Bond angles (˚)	1.5	1.5	1.5	1.5	1.5
Average pairwise r.m.s. deviation (Å)‡					
Protein (residues Met1-Ala53 of both subunits)					
Heavy	0.85 ± 0.12	0.99 ± 0.12	1.01 ± 0.16	0.96 ± 0.10	0.97 ± 0.10
Backbone	0.28 ± 0.03	0.49 ± 0.11	0.43 ± 0.10	0.45 ± 0.07	0.45 ± 0.09
Protein (residues Met1-Pro58 of both subunits)					
Heavy	1.11 ± 0.24	1.00 ± 0.12	1.26 ± 0.18	1.26 ± 0.22	1.16 ± 0.14
Backbone	0.71 ± 0.28	0.55 ± 0.11	0.89 ± 0.22	0.94 ± 0.32	0.78 ± 0.19
RNA (residues indicated bound to protein subunit A)	101-116	119-136	143-157	158-172	138-142
All RNA heavy atoms	0.41 ± 0.11	0.65 ± 0.23	0.50 ± 0.15	0.46 ± 0.12	0.53 ± 0.14
Complex (All heavy atoms in complex (C, N, O, P))					
Protein residues Met1-Ala53	0.76 ± 0.09	1.19 ± 0.27	0.90 ± 0.13	0.93 ± 0.10	0.93 ± 0.09
Protein residues Met1-Pro58	0.94 ± 0.17	1.20 ± 0.26	1.07 ± 0.14	1.13 ± 0.16	1.09 ± 0.12
Ramachandran statistics					
Most favoured regions	82.3 %	82.2 %	82.9 %	81.6 %	81.7 %
Additionally allowed regions	17.1 %	17.7 %	16.4 %	17.8 %	17.9 %
Generously allowed regions	0.2 %	0.0 %	0.7 %	0.6 %	0.4 %
Disallowed regions	0.4 %	0.1 %	0.0 %	0.1 %	0.0 %

Table S1: NMR and structure statistics.

*δ-angles in the loop residues: 50˚-110˚ for C3′-*endo* conformation and 130˚-190˚ for C2′-*endo* conformation.

†Protein backbone angles: the φ- and ψ-angles were determined using TALOS+ (26), RNA backbone angles: based on A-form geometry derived from high-resolution crystal structures: α=270˚-330˚, β=150˚-210˚, γ=30˚-60˚, δ=50˚-110˚, ε=180˚-240˚, ζ=260˚-320˚. The restraints were only used for double-stranded regions, which were identified by the presence of a protected imino resonance and confirmed by NOEs.

‡ Pairwise r.m.s.d was calculated among the ten lowest energy structures.

Table S2: All the RNA constructs used in this study are shown (from 5′ to 3′). The GGA binding motifs are highlighted in red.

Constructs from RsmZ RNA:

SL1: GG GUGUCGACGG AUAGACACCC

SL2: GG GCCA UCAA GGAC GAUG GU CC

SL3: GGG AUCGCAGGAA GCGAUCCC

SL4: GGGUCAUCAGG ACGAUGACCC

9nts-GGA$_{39\text{-}41}$: UCAGGAC AU

23nts-GGA$_{76\text{-}78}$: GAGAAAGGAACACAGAGACUAGG

GGA$_{term}$: GAGCUAGGGAAAAAUGUGGGCGGGUCAUACCGCCCCUUUUUUU

Constructs from *hcnA* mRNA 5′-UTR:

hcnA-GGA#1: GAGGGACCGGGUCGUUGCCCGGUCCC

hcnA-GGA#2: GAGACCGGGACGUUGCCCGGUCUC

hcnA-GGA#3: GAGCAUGGACGGCGAGACGCCGGGUA

hcnA-GGA#4: GAGCAUAGACGGCGGGACGC CGGGUA

20nts-RBS: GGGCUUCACG GAUGAAGCCC
